# Challenges in implementing opioid agonist therapy in Lebanon: a qualitative study from a user’s perspective

**DOI:** 10.1186/s13011-018-0151-8

**Published:** 2018-04-19

**Authors:** Ali Ghaddar, Sanaa Khandaqji, Zeinab Abbass

**Affiliations:** 1 Department of Biomedical Sciences, Lebanese International University, Beirut, Lebanon; 2 Observatory of Public Policies and Health, Beirut, Lebanon; 3 Department of Narcotics, Ministry of Public Health, Beirut, Lebanon; 4 School of Pharmacy, Lebanese International University, Beirut, Lebanon

**Keywords:** Buprenorphine, Qualitative, Opioid substitution therapy, Lebanon

## Abstract

**Background:**

Opioid agonist therapy (OAT) has been implemented for the treatment of individuals with opioid use disorders in Lebanon since 2011, but has not been evaluated yet. The aim of the study is to describe the implementation of the first pilot OAT program in Lebanon from the users’ perspective.

**Methods:**

Data collectors gathered data from male participants during June 2016-July 2016. Eighty-one out of 94 patients agreed to participate in the study. Data regarding access to treatment, satisfaction with the treatment protocol and treatment outcomes, patient-provider relationship, and misuse and diversion was collected through semi-structured qualitative interviews. Data saturation was reached after 81 interviews; once no new themes were reported.

**Results:**

Findings showed inequalities in access to treatment and showed that OAT improved mental and social wellbeing among users who had financial access and complied with the program protocols. Registering in the program protected users from arrest and reduced their economic burden. Among the main encountered challenges were fear of dependence to buprenorphine, restricted geographical access to treatment, misuse and diversion of buprenorphine.

**Conclusion:**

Results implicate inequalities in access to OAT as one important gap to be tackled in the management of OAT in Lebanon. Further research should be done in order to understand the challenges in the implementation of the program from the providers’ perspectives.

**Electronic supplementary material:**

The online version of this article (10.1186/s13011-018-0151-8) contains supplementary material, which is available to authorized users.

## Background

The use of buprenorphine for opioid agonist therapy (OAT) was officially approved in Lebanon in 2011. A small-scale take-home pilot OAT program has been in implementation ever since, with around 1800 enrolled patients (800 of which are currently active). The national guidelines adopt take-home buprenorphine prescribed by authorized psychiatrists with weekly monitoring for adherence and response to treatment in clinical settings.

Buprenorphine is a mixed opioid agonist/antagonist used for opioid detoxification, and as a substitution therapy to prevent withdrawal symptoms in inpatient and outpatient settings [1]. With lower abuse potential than other full opioid agonists, office-based treatment is considered an effective and safe agent to use in OAT [2]. Due to its mode of action as a partial agonist, limited respiratory depression, and its ceiling effect, buprenorphine is highly effective in reducing overdose [3]. In France, overdose incidence decreased by 79% since the introduction of buprenorphine [4]. High-dose buprenorphine is also effective in suppressing illicit opioid use [5]. Compared with observed induction, take-home buprenorphine has advantages in terms of its feasibility, comparable safety, and fewer logistic barriers [6, 7].

Nevertheless, emerging evidence revealed certain problematic issues related to buprenorphine, including its potential diversion and misuse through injection [8]. In Australia, buprenorphine diversion showed 10 times higher than methadone [9]. Buprenorphine represents the most commonly abused drug in several countries such as Finland [10], and is illicitly used (mainly intravenously) in a wide variety of settings, including France [4], Sweden [11], Norway [12], Ireland [13], and Spain [14]. Illicit use of buprenorphine raised concerns in India, being the most commonly injected drug after heroin [15] and being the initiating injecting drug for many IDUs [16]. Diversion of buprenorphine is also widespread in South Asia [17].

The qualitative assessment of patient’s perception about the received care in addiction treatment services is frequently used as an indicator of the quality of care and of the use and the benefits of the treatment [18–20]. Moreover, user’s satisfaction in addiction treatment programs has been also related to positive treatment outcomes and better retention in the treatment [21, 22].

Six years have passed since the start of implementation of the pilot OAT program in Lebanon. In this program, patients diagnosed with opioid use disorder according to DSM-V criteria are registered, and receive weekly take-home buprenorphine from the centralized dispensing unit of the Ministry of Health. The monthly cost of treatment ranges from 85 to 250 USD. Registered patients are followed-up on a weekly basis by multidisciplinary teams of treatment providers, comprising of a psychiatrist, psychologist, social worker and a nurse, all experienced to work with drug users. The weekly follow-up visits to the treatment centers involve psychosocial support and monitoring for misuse and stability of response.

A quantitative evaluation of the OAT program in Lebanon has recently been conducted [23]. Although the program produced positive outcomes on quality of life, mental and social wellbeing and reduction of risky behavior among users, very low retention rates has been observed. The same study pointed out to possible information bias related to overestimation of the outcomes and finally called for the importance of exploring the predictors of adherence using qualitative assessment.

On the other hand, there has been no independent evaluation of the challenges related to the program’s setup and its implementation and to its degree of adaptation to the needs and culture of persons seeking treatment from opioid dependence disorder in Lebanon.

The objective of the study is to provide a qualitative assessment of the OAT program in Lebanon from a user’s perspective, and to describe how inequalities in the access to treatment could shape users’ compliance and perceptions about the treatment outcomes*.*

## Methods

### Study design

This qualitative study was conducted at one of the two OAT dispensaries in Beirut, Lebanon. Female participants were not included, as most of the OAT users in Lebanon are males. Also, participants registered in the program for less than 3 months were excluded. All patients who were found in the dispensary were included in the study. Only male patients were included as most of OAT users in Lebanon are males. Participants with less than 3 months in therapy were excluded.

Eighty-one (86%) out of 94 approached patients agreed to participate. Non-respondents were offered to be interviewed at a different time, one accepted and was interviewed the following week, and the others excused for having no time.

### Ethical consideration

Ethical approval was obtained from the Institutional Review Board of the Lebanese International University (ethical approval: Ref: LIUCRE-140320-1). Verbal informed consent was obtained from all participants. All interviews were audio-recorded except four, as participants had refused to be audio-recorded. In this case, written notes were taken instead.

### Data collection

Data was collected during 15 consecutive days between June 2016 and July 2016. by three data collectors experienced in qualitative research. The majority of interviews conducted with OAT patients were done by two data collectors (first and second authors). They approached participants in the alley outside the dispensary, to avoid any direct contact between them and the healthcare providers. Patients were asked to participate in the study after being briefed about its objectives. The third data collector (AT) was an OAT patient specially trained for the study purpose. He interviewed patients outside the hospital, as some of them were not officially registered and were self-treating with diverted buprenorphine. Interviews were done using a semi-structured guideline and lasted around 20 min. Information was gathered anonymously about access to treatment, satisfaction with treatment rules, patient-provider relationship, perceived benefits and side effects of the drug, and misuse and diversion (guideline as online supplement). Questions included: *In general, are you satisfied with your achieved outcomes regarding family relations, self-image, social functioning, avoiding arrest and reducing financial burden? Have you sold or given buprenorphine to peers? Which part of the treatment/ rules do you find hardest to meet?*

### Data analysis

The theoretical sampling method with selective coding was used in this study in order to formulate new emerging theories from collected data [24]. Selective coding took into consideration the concepts found in previous studies related to the topic. The grounded theory with constant comparison was adopted in order to develop a theory as it emerges [25]. As codes were developed during reviewing the transcripts for relevant emergent themes, data was simultaneously analyzed in order to decide what data to collect in the next step. Themes were identified from the codes drawn out from the transcripts, reviewed and analyzed in order to identify structures that helped construct the theoretical model. The theory induced from the data analysis was then applied to existing data in order to assess their validity.

Data saturation was reached after 81 interviews when no new facts or ideas were likely to appear in any of the categories, the categories are well developed in terms of their properties, dimensions and variation, and the relationship between categories are well established and validated [24, 26, 27].

The socio-demographic characteristics of the participants are summarized in Table 1. Participants were males with average age 29.1 years (range: 20–56 years). The majority were single and had no stable income. The average time spent in treatment was 2.40 years (range: 1 month - 6 years). More than half of the participants (58.0%) had ever injected drugs and 45.7% ever injected buprenorphine. 12.3% were currently injecting buprenorphine and 37.0% mixed buprenorphine with other drugs.

**Table 1:** Socio-demographic characteristics of the participants

*Age (years)*	*n* (%)
18-25	36 (44.4%)
26-30	22 (27.2%)
31-35	9 (11.1%)
36+	14 (17.2%)
*Educational level*	
Primary school education	38 (46.9%)
Secondary school education	43 (53.2%)
*Currently employed*	
Yes	33 (40.7%)
No	48 (59.3)
*Stable income*	
Yes	49 (60.4%)
No	32 (39.6%)
*Marital status*	
Single	67 (82.7%)
Married	14 (17.3%)

## Results

Findings revealed the existence of inequalities in the access to treatment that shaped users’ compliance to therapy and their satisfaction with its outcomes. Two different views regarding the outcomes of therapy emerged. The first group of participants (G1) did not express major financial or logistic concerns in their access to therapy. All 52 participants who belonged to this group were OAT registered users who have been in therapy for at least one year. The majority of them were not using illicit drugs along with buprenorphine and none reported current injecting drugs. The other group (G2) (29 participants) had clear financial barriers to access therapy. Almost all of them were unemployed and unregistered users who occasionally got access to diverted buprenorphine form the black market. Others were officially registered users in the OAT program who had to interrupt their therapy for being unable to afford its cost or for deciding to self-treat their addiction without medical supervision. All participants who belonged to this group currently use illicit drugs together with buprenorphine and many of them currently injected buprenorphine.

### Perceived benefits

Some participants reported improvements in different domains of mental and social wellbeing during therapy and others expressed concerns and challenges related to therapy (Table 2).

**Table 2:** Main reported dimensions of improvements and concerns related to therapy

	*Users with good access to therapy (G1) (N = 52)*	*Users with limited access to therapy (G2) (N = 29)*
*Reported improvements*	*n (%)*	*n (%)*
Physical health	37 (71.2%)	8 (27.6%)
Mental health	39 (75.0%)	11 (37.9%)
Reducing drug cravings	41 (78.9%)	4 (13.8%)
Abstinence from other drugs	33 (63.5%)	2 (6.9%)
Social functioning	42 (80.8%)	12 (41.4%)
Family relations	34 (65.4%)	14 (48.3%)
Self-image and confidence	44 (84.6%)	11 (37.9%)
Avoiding arrest	47 (90.4%)	26 (89.7%)
Reducing financial burden	50 (96.2%)	20 (69.0%)
*Reported concerns*	*n (%)*	*n (%)*
Dependence to buprenorphine	29 (55.8%)	21 (72.4%)
Treatment side-effects	9 (17.3%)	19 (65.5%)
Geographical access	7 (13.5%)	4 (13.8%)
Time and other logistic constraints	11 (21.2%)	8 (27.6%)
Treatment cost	6 (11.5%)	27 (93.1%)
Difficulty to adhere to treatment protocol	13 (25.0%)	19 (65.5%)

Most participants who belonged to G1 (37 participants) were generally satisfied with the impact of the treatment on physical and mental wellbeing. They considered that OAT is effective in reducing illicit opioid use and its associated craving:

–*“It does not make you nostalgic…under heroin, I need to inject every few hours, but now, the morning tablet and that’s it. Many people I know managed to quit and stood back on their feet, this encouraged me. After the tablet, you have no desire to take anything else. Before, I took everything available”–* (M3, 35 y/o, G1)

Interviews revealed the importance of OAT in sustaining users’ social inclusion, especially among participants of G1. They considered that OAT enhanced their self-confidence, their workability and their image in society. Several quotes were selected to illustrate this perspective:

*–“Before therapy I was excluded from society; I did not wear decent clothes, I dressed like an addict and didn’t shower. All I cared about was how to obtain H [heroin]. Now, I feel respected at work and in society…”–* (M1, 32 y/o, G1)

*–“I don’t have to lie to them anymore… nobody trusted me before, although I wanted to behave good and loved my fiancée, I frequently forgot everything about her when my friends called me to inject”–* (M5, 32 y/o, G1)

*–“At least you don’t have needle marks… I can wear short sleeves. I feel more energetic, I can concentrate at work” –* (M27, 24 y/o, G1)*.*

It was also obvious from interviews with participants of G1 that OAT helped users avoid drug scenes and the negative influence from peers. Some quotes were selected to explain this idea:

–“*It takes you out of the street… before, I was always searching for H in neighborhoods… they [drug dealers] are always there, offering you H and you cannot refuse”* – (M16, 20 y/o, G1)

–*“We decided to register in order not to remain on the street with the bunch of heroin addict hooligans”–* (Father of M7, 19 y/o, G1)

–*“Instead of waiting all day for drug sellers in the streets, I can just come here to take my drug” –* (M9, 28 y/o, G1)

Furthermore, it appeared that in G1, the treatment promoted family acceptance and strengthened family support. The following quotes support this viewpoint:

*–“It is completely different; my family supports me financially to get the drug and I feel more protected”–* (M16, 20 y/o, G1)

*–“I am no family enemy anymore, my parents stopped insulting me and making me feel undesired. They believe in me and we now do things together”*– (M34, 25 y/o, G1).

On the other hand, participants who belonged to G2 experienced constant withdrawal symptoms and had important concerns related to the benefits of OAT.

### Perceived concerns

#### Dependence to buprenorphine

Both groups of users (with and without good access to OAT) were concerned about the long-term nature of the therapy and about its social acceptance. Participants of G1 perceived OAT as *“switching from one type of addiction to another”* upon realizing their unexpected prolonged time duration of the therapy. Apparently, many initially saw in the OAT program a way out of their addiction. More than half (62%) declared not having been explicitly briefed about the long duration of maintenance therapy upon joining the program and expected a better outcome and shorter duration of therapy. The following quotes elaborate this point:

*–“What is the point, they sell us this legal drug, and people get attached to it… I mean until when I will keep buying this drug? I need a solution”*– (M11, 29 y/o, G1).

–*“I was enthusiastic at first, believing that this is the ultimate solution, but I realized it is not possible to quit… I tried; you just can’t”–* (M48, 31 y/o, G1)

*– “When you get registered, they just recommend this therapy as an option, and you get to register without really being explained that it will last forever, nobody tells you that from the beginning” –* (M4, 26 y/o, G1)

They clearly viewed the long-term use of buprenorphine as socially unacceptable and had concerns about dependence. Some of the below quotes illustrate this perspective:

*–“I cannot imagine my life going to the dispensary and all the complications for my entire life. Imagine my kids asking me what is this drug … it is difficult to explain” –* (M2, 27 y/o, G1)

*–“People will still look at you as an addict, it will chase you for your entire life. The only difference is that now I take a legal drug whereas I had to search for H in the streets”*– (M39, 28 y/o, G1)

The majority of participants who belonged to G2 (72.4%) expressed the same concerns related to dependence on buprenorphine, especially after experiencing the severe associated withdrawal symptoms. Those were completely convinced that stopping buprenorphine is just impossible, as described by the following quotes:

–*“You cannot stop a single day, you feel extremely exhausted. The consequences are more awful than the brown [heroin]… I get feelings of knives stabbing inside my stomach… you cannot walk, you have to drag your legs…”–* (M54, 27 y/o, G2)

–“*It just takes you from one problem to another, from the dependence on heroin to the dependence on buprenorphine*”– (M60, 32 y/o, G2)

–*“We will die taking the drug…it is like blood pressure drugs”–* (M73, 40 y/o, G2)

*–“Had I known previous to starting this program that it will be forever, I wouldn’t have registered”* – (M54, 27 y/o, G2)

Some expressed serious concerns about this issue, especially when they have trouble securing the treatment cost. One participant explained:

–*“The treatment is temporary, and when the time comes and I cannot afford it, I will go back to the streets”* – (M65, 25 y/o, G2)

### Financial and geographical access to treatment

Almost all participants in G1 (93.8%) agreed that their engagement in the therapy lowered their financial burden:

–*“Before, I needed to pay $50-100/day for a couple of grams of heroin, while now it costs me just $3/day for my 2 tablets of buprenorphine, you see” –* (M23, 21 y/o, G1)

–*“I lost all $50,000 in two years when I was using heroin, now I can easily afford to pay for the tablets” –* (M37, 22 y/o, G1)

However, participants who belonged to G2 had difficulties in securing the weekly therapy cost, especially when the majority of them were unemployed. One participant expressed briefly the concerns of others:

–*“When you are not working, it is not easy. The pressure on me is huge, the LBP 70,000/week (around $50) for buprenorphine, transportation, and laboratory tests …many times I beg friends for money”* – (M58, 42 y/o,, G2)

Living outside Beirut appeared to be an important barrier to access therapy. Almost all participants lived in Beirut and its suburbs. The two interviewees from North Lebanon (85 km away from Beirut) had private cars but mentioned that numerous of their peers have no means to reach the dispensaries (one located in Beirut center and the other 15 km Northern Beirut). Many participants residing in Beirut also considered transportation a barrier to access:

*–“I feel sorry each time my father has to leave work to take me get the drug”-* (M54, 27 y/o, G2)

### Avoiding arrest is a main motivation to register

Perhaps one of the most commonly reported treatment benefit is the fact that it reduced the risk of arrest:

–“*We do not fear police anymore, with prescription papers, you feel secure when going out”–* (M18, 32 y/o, G1)

The discourse revealed that many participants in G2 hide behind their prescriptions and do not refrain from using illicit drugs:

*–“All those people you see [pointing out to the direction of registered patients at the OAT dispensary] share their tablets for the exchange of a bit of heroin. Not only me, many still use heroin at least twice a week. We just register to be on the safe side, not to run out of drugs, to avoid trouble with the police. But it is quite impossible to quit, this substance is crazy”* –(M84, 28 y/o, G2)

*–“Buprenorphine is legal heroin”*– (M61, 31 y/o, G2)

–*“Before, every other week I had to take the bus to Baalbek [North Lebanon] to get heroin, and on the way I got arrested twice…the road is too risky, they wait to arrest you”–* (M67, 26 y/o, G2)

### Injecting drugs and buprenorphine misuse

Maintenance therapy reduced injecting behavior. The percentage of participants reporting injecting drugs reduced from 58% to 12% after therapy. This discussion led to the emergence of a new theme about the misuse of buprenorphine mainly by injecting in both G1 and G2. 45.7% of the participants reported having previously injected buprenorphine and 12.3% reported current injection:

*–“The moment I do my urine test I have my heroin equipment ready to inject”–* (M18, 32 y/o, G1)

*–“Every time I stop and see my sister injecting heroin I try again”–* M76, 30 y/o, G2)

However, almost all participants mentioned that most of their peers currently inject buprenorphine:

–*“99% of people in the program are hitting buprenorphine”–* (M90, 38 y/o, G2)

The discourses suggested that injecting buprenorphine is the preferred mode of administration for many participants. The following quotes elaborate this perspective:

–*“It gives a sweeter effect and it lasts longer and so I mean why not inject? This is the first time I hear that it is harmful…”* – (M88, 35 y/o, G2)

–*“Injecting buprenorphine makes more sense”*– (M21, 27 y/o, G1)

–*“When you hit, it makes you contain yourself. With only half a tablet, you need nothing else until the next day”* – (M49, 22 y/o, G1)

–*“It is so difficult to cease injecting. It is a virus inside”–* (M18, 32 y/o, G1)

*–“My brother died at hospital after his leg inflamed, I knew it was due to injecting…I know it is evil, still I prefer to inject buprenorphine, there is no other way that keeps me standing”* – (M49, 22 y/o, G1)

### Diversion

Participants who were unregistered in OAT program (15%) were at the dispensary site for trying to obtain diverted buprenorphine from registered peers. The below discussion highlight the context of diversion of buprenorphine in the black market:

*–Interviewer: “I can’t figure out why someone would sell his medication?”*

*–Participant (M75): “some of them need money to buy their medications and thus they share”*

*–Interviewer: “but then the therapy might not work well, if they don’t adhere as prescribed, right?”*

*–Participant: “what else to do, you can manage with one tablet- I myself sold half of my tablets”*

*–Interviewer: “and you were fine? Why did you unregister in the program?”*

*–Participant: “if you need the money you will sell, you have to manage at the end, they unregistered me because I always had difficulties, the visits and all the strict control”*

*–Interviewer: “so you mixed other drugs during the treatment?”*

*–Participant: “when I am out of Bup. I searched for heroin from time to time, but I was Ok”.*

Further accounts confirmed that prescribed buprenorphine was diverted:

–*“I can do with one tablet/day and sell the rest to secure the cost of buprenorphine. Some indifferent people sell the tablet (for around $15-20) to buy other drugs”* – (M93, 38 y/o , G2)

Participants generally did not acknowledge diverting buprenorphine (only 14% reported diversion). However most of them declared having been frequently requested to share or sell their tablets:

–“*Right outside the dispensary, they tire me out ‘man, please’, and all that… how much do you want for it?’ and ‘just one tablet’, but I don’t sell at all, I need it more than anyone else does”* – (M32, 30 y/o , G1)

One participant highlighted the main reason that made him refrain from registration, and is instead using diverted buprenorphine:

*–“I cannot register in the treatment because I work and I do not want my employer to know. I do not want to get fired”–* (M59, 28 y/o, G2)

Another explained the logistic barriers supposed by engaging in the program:

*–“I prefer not to register because it is practically impossible to drive up to the NGO every week to do urine tests”–* (M78, 30 y/o, G2)

It is important to note, however, that a few of them had previously registered in the program, but had failed to adhere to the treatment protocol for financial or logistic reasons:

–*“The program is not flexible”* and

–*“I was excluded for missing three consecutive follow-up sessions”*– (M51, 27 y/o, G2)

These patients found a way to get access buprenorphine without registering in the treatment:

–*“Buprenorphine is a savior when short of money to buy heroin”–* (M70, 32 y/o, G2)

## Discussion

This study aimed to provide a qualitative assessment of the pilot OAT program in Lebanon from the perspective of the users. Results revealed inequalities in the access to treatment and showed that OAT generally improved mental and social wellbeing among users who had financial means to access the program and managed to comply with the treatment protocols. Registering in the program protected users from arrest and reduced their financial burden. Challenges include fear of dependence, restricted access to treatment and misuse and diversion of buprenorphine.

Results provide several indications to improve patient-provider relationship. Firstly, the common observed concern about dependence on buprenorphine could be resolved through providing patients with adequate briefing about the long-term nature of buprenorphine treatment and its properties that allow gradual withdrawal without big discomfort in comparison to methadone [28]. In fact, promoting transparent communication between providers and patients shape patients’ believes and expectations, and in turn influence patient satisfaction and the achievement of positive treatment outcomes [29].

Results replicate those found in previous studies and identified the main motivations for diversion and illicit use of buprenorphine as self-detoxification and lack of financial means to formally register in the treatment [12, 30]. The fact mentioned by one interviewee that *“many of us register in the program because buprenorphine is less expensive than heroin”* has been reflected upon in a previous study in Australia [31]. In fact, buprenorphine diversion has been partly attributed to its feasible access to IDUs with limited income [14]. However, this fact has not been contrasted in middle and low development settings. Buprenorphine diversion, though frequently previously reported in various contexts, raises concerns for its potential of use by individuals initiating opioids and/or injecting drugs, as indicated in previous studies [12, 17, 32]. Injecting buprenorphine is also not new and intravenous use represents the most efficient route of administration in terms of bioavailability where euphoric effects could be obtained with relatively small doses [33]. The motivation to engage in OAT as a way to avoid arrest and police harassment has also been mentioned in previous studies [34].

### Limitations, strengths and future research

It is worth mentioning that data of OAT patients from all clinics that provide OAT in Lebanon are connected through a central network to a central database in the Ministry of Health. This system has the advantage of tracking patients IDs in order to prevent duplication of services and *doctor shopping*, a phenomena common in other contexts [35]. The study has certain limitations related to the sample. Results do not reflect the opinion of female OAT patients. Moreover, it would have been interesting to include patients from the other OAT hospital dispensary to compare findings.

The current study also has several strengths. It actually provides the first qualitative overview about the opinion of users about the OAT program in the Middle East and North Africa (MENA) countries, excluding Iran. Moreover, participants were selected from one of the only two available dispensary centers in the country. Information bias was also reduced through coding data by two different researchers who discussed disagreements whenever present. During analysis, results were validated with other research team members including previous IDUs. Results were consistent with those presented in previous studies and support their transferability to other MENA countries.

Future research is warranted to understand the reasons of injecting buprenorphine from a social perspective rather than clinical pharmacological effect evaluation. The public health and economic implications of injecting buprenorphine should be explored rather than understanding the pharmacological effects of the drug mode of administration. Future research should be done in order to understand the challenges in the implementation of the program from the providers’ perspectives.

## Conclusion

Results revealed that the OAT program in Lebanon should be supported and highlighted certain gaps that should be tackled to improve its implementation. Results highlighted the importance of continuing the efforts to advocate for better social tolerability of OAT in Lebanon, strengthening the outreach and awareness activities to cover unregistered opioid-dependent participants and to achieve a better communication between providers and users. Finally, the observed inequalities in the access to treatment call for the need to scale-up the program and widen geographical access to districts other than the capital Beirut and to the most marginalized population groups with low socio-economic status.

## Additional file

Additional file 1: GUIDELINE_Patients. (DOCX 24 kb)
