# Supplementary material for: Challenges in implementing opioid agonist therapy in Lebanon: a qualitative study from a user’s perspective
Source: Subst Abuse Treat Prev Policy. 2018 Apr 19;13:14. doi: 10.1186/s13011-018-0151-8 (PMC5909215; doi:10.1186/s13011-018-0151-8)
Supplement: Supplementary file 1 — GUIDELINE_Patients. (DOCX 24 kb) [file 13011_2018_151_MOESM1_ESM.docx]

| **Data collector:** | **Data collection site:** |
| --- | --- |
| ***Socio-demographics*** | |
| Gender | Age |
| Marital Status | Place of living |
| Work status | Previous imprisonment |
| Income | Previous residential treatment/ detoxification program |
| ***Registration status*** | |
| Are you currently registered in the OST program?  *In case not:* Have you been ever registered in the OST program? For how long? Why did you quit? |  |
| *In case yes:* Ask below |  |
| How long have you been taking buprenorphine? |  |
| Who is your prescribing physician? |  |
| In which center/NGO you are receiving follow-up? | *Probe*: Private clinic or NGO? |
| What is your prescribed dose? Have you tapered down the dose? |  |
| Have you been registered in another center before? Specify |  |
| ***Access to the program and treatment rules*** | |
| Why did you decide to get enrolled in the program? | *Probe*: a) desire to improve health; b) desire to end dependence on illegal drugs for good; c) desire to improve relation with family and social wellbeing; d) avoid arrest |
| Was it easy to find a doctor to prescribe buprenorphine? | *Probe*: geographical distance |
| Did you set with your physician long term expectations and goals at the time you have entered the program? |  |
| Can you afford to pay the treatment cost? |  |
| Were you well informed about treatment alternative before you started the treatment? Was it you or the physician who explicitly suggest buprenorphine as first alternative? | *Probe*: Other drug? Detoxification, other residential rehabilitation program? |
| Which treatment rules do you find hardest to meet? | *Probe*: a) Stopping all illegal drug use; b) maintaining abstinence on the long term; c) Dose supervision; d) Tapering dose over time; e) commitment to attend all appointments; f) commitment to undergo psychosocial counseling; g) urine tests, h) not injecting |
| What conditions would have encouraged you to start treatment earlier? | *Probe*: a) better flexibility in the rules; b) better geographic availability; c) lower financial cost; d) more information about the options of treatment; e) less conditions to start treatment |
| ***Patient provider relationship & satisfaction*** | |
| Are you satisfied with your achieved outcomes from the program? |  |
| How do you judge your physical health? |  |
| How do you judge your mental health? |  |
| Are you satisfied with the program in terms of avoiding arrest and the relationship with low enforcement personnel? |  |
| Are you satisfied with the staff working in the center who provide counseling and follow-up? | *Probe:* With each of the nurse, social worker, physician, and psychologist. |
| Are you satisfied with the staff working in the dispensing unit? |  |
| Would you recommend the program to others? |  |
| Are you satisfied with the eligibility criteria to enter or remain in the program? |  |
| Are you satisfied with the dose prescribed? Have you tried to taper down the dose? If yes, was the taper down with the physician approval? |  |
| Are you satisfied with the pain management program? |  |
| Are you satisfied with the clinic schedule? |  |
| Are you satisfied with the location access? |  |
| Do you have any concerns about stigma in the clinics NGO? |  |
| Are you satisfied with the financial access? |  |
| Are you receiving psycho-social and behavioral counseling? Is this being helpful for you? |  |
| **Precipitated withdrawal symptoms** | |
| - Are you experiencing physical and emotional symptoms such as agitation, anxiety, insomnia, excessive yawning, muscle aches, runny nose, watery eyes, abdominal cramping. | *Probe*:  Are you dissatisfied with buprenorphine for these undesired physical and emotional symptoms? |
| Are you taking another drug with buprenorphine?  Have you tried talking to the doctor?  Have you tried to stop other opioids to avoid these symptoms?  Alone or with medical supervision? | Check full opioid agonists  Including heroin, prescription painkillers like OxyContin or Percocet, benzodiazepine |
| ***Misuse & precipitated withdrawal symptoms*** | |
| Are you currently taking illicit drugs in addition to buprenorphine? | *Probe*: a) Specify which drug; b) how frequent per month; c) check the full opioid agonists including heroin, prescription painkillers like OxyContin or Percocet, benzodiazepine |
| - Are you experiencing physical and emotional symptoms such as agitation, anxiety, insomnia, excessive yawning, muscle aches, runny nose, watery eyes, abdominal cramping. | *Probe*:  Are you dissatisfied with buprenorphine for these undesired physical and emotional symptoms?  Have you tried talking to the doctor? Have you tried to stop other opioids to avoid these symptoms? Alone or with medical supervision? |
| If yes, specify the reasons behind taking illegal drugs in addition to buprenorphine | *Probe*: a) desire to get high occasionally; b) buprenorphine not controlling craving very well |
| Have you ever injected buprenorphine? Are you currently injecting buprenorphine?  How often? |  |
| Have you experienced side effects related to injection? | *Probe:* acute limb ischemia, emboli, sepsis, endocarditis, infection, etc. |
| Have you ever snorted buprenorphine?  Are you currently snorting buprenorphine? How often? |  |
| What are the main reasons for injecting/snorting buprenorphine? | *Probe*: a) Social acceptability of injecting buprenorphine; b) sublingual buprenorphine not controlling craving very well; c) opinion about the sufficiency of the prescribed dose is not sufficient; d) effect of injecting on euphoria, etc. |
| Was buprenorphine your first initiating injecting drug? |  |
| Compare injecting buprenorphine with injecting heroine?  Explain from the clinical and social perspectives | *Probe:* effect on euphoria, pain reduction, anxiety reduction.  Effect on society: are you more comfortable to face society, parents? |
| Have you ever shared syringes? Was this before taking buprenorphine?  Are you currently sharing syringes? |  |
| Diversion | |
| Have you sold or given buprenorphine to peers? Specify the reasons |  |
| Have u ever been forced to sell your medication? |  |
| From where do you obtain buprenorphine? |  |
| Why you are not registered? | *Probe*: a) cannot afford the cost; b) to avoid stigma; c) lack of trust in the healthcare workers/ NGO; d) strict criteria to maintain enrollment; e) logistic barriers (transportation, others) |
| Are you aware if registration is confidential? |  |
| Do you believe that available systems will protect really your privacy and confidentiality as stated in their regulations? |  |
| Do you consider bup use as a legal use of drugs with no other additional benefit? |  |
| To what extent you think your shifting from one type of addiction (opioid) to another type (bup) |  |
